# Using a Slit to Suppress Optical Aberrations in Laser Triangulation Sensors

**DOI:** 10.3390/s24082662

**Published:** 2024-04-22

**Authors:** Steven Pigeon, Benjamin Lapointe-Pinel

**Affiliations:** Département de Mathématiques, Informatique et Génie, Université du Québec à Rimouski, Rimouski, QC G5L 3A1, Canada; benjamin.lapointe13@gmail.com

**Keywords:** laser, laser triangulation sensor, diffraction pattern, optical aberrations, image sensor, range sensing, image analysis

## Abstract

In this paper, we present a laser triangulation sensor to measure the distance between the sensor and an object without contact using a diffraction slit rather than a traditional lens. We show that by replacing the lens with a slit, we can exploit the resulting diffraction pattern to have finer and yet simpler image analysis, yielding better estimation of the distance to the object. To test our hypothesis, we build a precision position table and a laser triangulation sensor, generate large data sets to test different estimation algorithms on various materials, and compare data acquisition using a traditional lens versus using a slit. We show that position estimation when using a slit is both more precise and more accurate than comparable methods using a lens.

## 1. Introduction

In many different applications, we are interested in measuring the distance between an object and a sensor without direct physical contact. When great distances are involved, the preferred approach is to use time-of-flight methods, in which a laser pulse is emitted and the time taken for the reflection to come back to the sensor is measured [1,2,3,4]. These time-of-flight sensors were pioneered in satellites [5] and are now often used for airborne exploration of regions covered in dense vegetation or that are otherwise inaccessible (for examples, see [6]). The accuracy of time-of-flight sensors is typically on the order of a few centimeters [7]. Acquiring distances with centimeter precision from time-of-flight sensors requires timing accuracies well under nanoseconds, as light travels 29.9792458 cm in one nanosecond. However, when time-of-flight is aided by other techniques, such as phase shift detection, accuracies on the order of 25 μm can be achieved ([1], Table 5), but this requires even more sophisticated, and therefore presumably even more expensive, hardware.

Laser triangulation sensors are commonly used when the distances considered between the sensor and the objects of interest are on the order of a few tens of centimeters or up to a few meters. Many of these sensors have accuracies on the order of micrometers [8,9,10,11,12,13,14]. Laser triangulation sensors are easier and less expensive to build than time-of-flight sensors, but they rely on image analysis algorithms to achieve precision rather than precision timing hardware.

**Figure 1:**
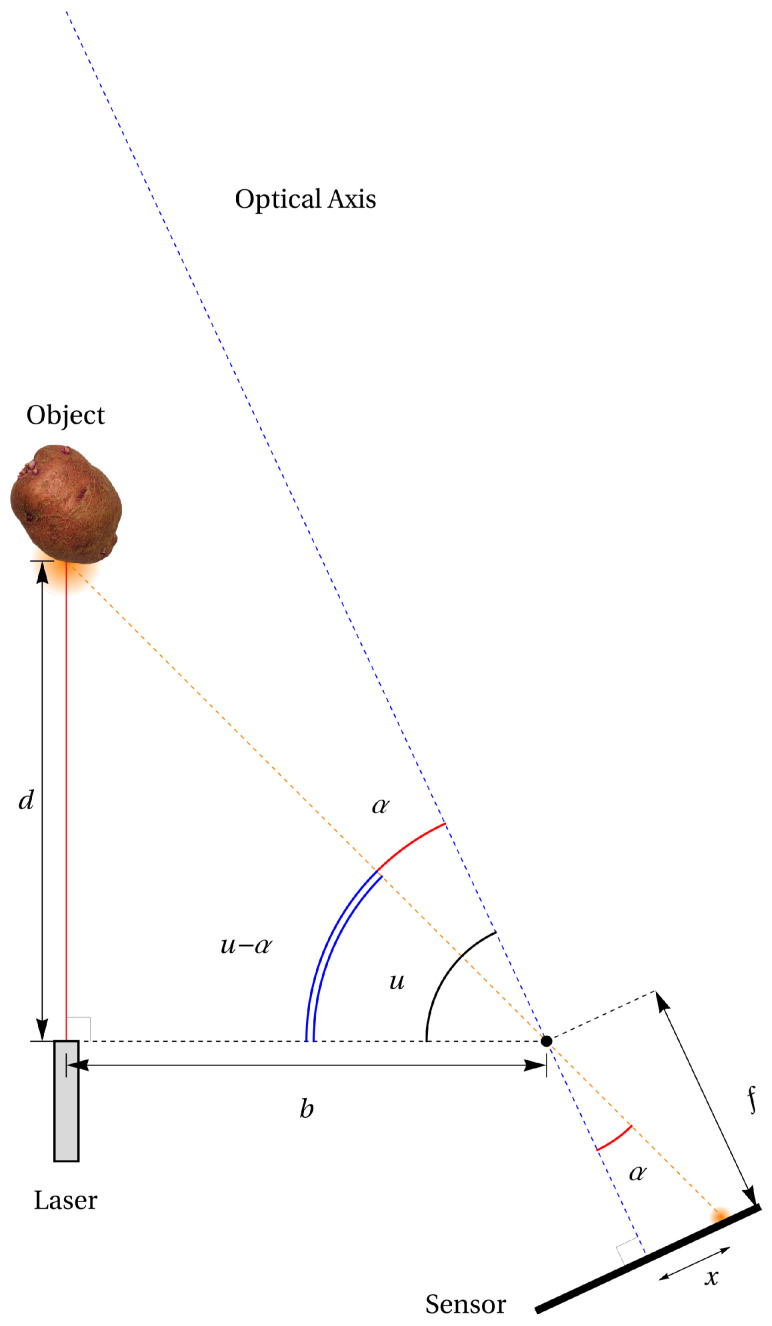
Laser range finding using triangulation.

In a laser triangulation sensor, a laser beam and the optical axis of a photosensitive sensor are placed at an angle, as schematized in Figure 1. In many applications, it is desirable to have the laser beam perpendicular to the sensor’s casing, which leaves the optical assembly to be placed with a certain angle, which is chosen so that the optical axis (or, loosely, the center of the field of view) corresponds to the center of the useful range of the sensor. As the laser beam reaches the object, it is reflected, and its reflection is projected against the photosensitive sensor through a lens or some other optical assembly. Here, we make the simplifying assumption that the laser beam and the optical axis lie in the same plane, and that this plane corresponds to one of the photosensitive sensor’s axes, which will ensure that the reflected laser spot is seen as moving only along that axis—horizontally, in our case—across the sensor. We can then estimate the angle of projection α using the focal length *f* of the lens (or its equivalent) and the center of the projection *x*; the problem is, therefore, to obtain an accurate estimation of *x*, which is the center of the projection. Knowing the angle α, the distance *b* between the laser source and the center of the optical assembly, and the angle *u* of the assembly relative to the casing, we can determine the angle u−α and, therefore, the distance *d* of the target relative to the sensor.

The angle α of the projection in the optical assembly is given by

$$\alpha =\tan^{-1}\left(\frac{x}{f}\right)\;,$$

and the distance *d* is found to be

(1) $$\begin{array}{cc} d & =d_{0}+b\tan\left(u-\alpha \right)\\ \; & =d_{0}+b\tan\left(u-\tan^{-1}\left(\frac{x}{f}\right)\right)\;, \end{array}$$

where $d_{0}$ is an additional offset that takes into account the sensor casing thickness and other assembly variations. A first calibration procedure would be performed at the moment of assembly, and subsequent calibrations would likely by performed periodically over the sensor’s lifetime in order to compensate for any mechanical changes due to temperature, vibrations, or other mishaps—none of which were tested for this proof-of-concept.

The preferred embodiment for the optical sensor in a laser triangulation system consists of a lens that focuses the image of the reflected laser onto a light-sensitive sensor: typically a CMOS or CCD image sensor [15,16,17].

However, as shown in Figure 1, the optical axis intersects the laser at an angle, which means that the focal plane of the lens, which is perpendicular to the optical axis, only provides a narrow region where the reflection of the laser is in good focus, as the lens usually has a limited depth of field. To mitigate this problem, it has been proposed to use so-called tilt-shift lenses to rotate the image plane in order to have the laser line lying entirely in the focal plane according to the Scheimpflug principle [11,18]. Traditional SLR and other fixed-sensor cameras require specialty tilt-shift lenses, but in a laser triangulation sensor, we are free to change the angle of the image sensor within the device, independent of the optical axis.

**Figure 2:**
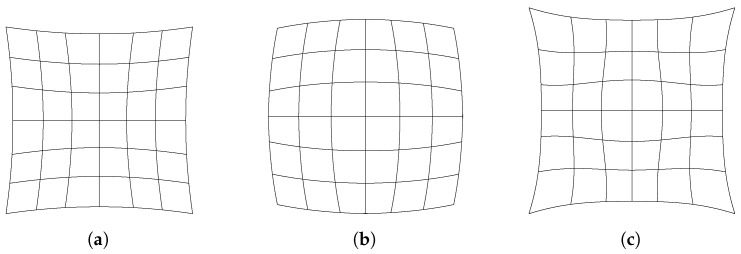
Typical optical distortions. (**a**) Pincushion. (**b**) Barrel. (**c**) Mustache.

Even if we suppose that we are able to use a tilt-shift lens or change the angle of the sensor to have the desired image plane, we are still bound to the limitation of the lenses. Indeed, lenses are subject to a number of optical aberrations. If the lens is spherical, it will show what is called spherical aberration wherein different regions of the lens will have different focal points, resulting in a “soft” or diffuse focus. If the lenses have different horizontal and/or vertical curvatures, they will suffer from astigmatism, resulting in images that are clearer in one direction than the other. A lens may exhibit coma aberration, wherein parallel rays entering the lens at an angle have different focal points, resulting in a comet-like projection of points—thus, the name. The lens can also show a number of other geometric distortions such as pincushion, barrel, or a combination of both, termed “mustache”, as shown in Figure 2, which warp the projected image and therefore introduce imprecision. Additionally, all lenses are subject to chromatic aberration, wherein rays of different wavelengths are refracted at different angles, resulting in images with a rainbow effect radial to the center of projection, typically with red fringes towards the edge of the image and blue fringes towards the center. However, chromatic aberration can be safely ignored since we will use a monochromatic light source: a laser.

Although not technically an aberration, lens flare, mostly internal reflection wherein light bounces around off the sensor and other optical elements, will typically produce one or several localized rings or circles of light in the image and, if sufficiently intense, can even cause diffuse internal illumination, known as glare, that will “wash out” the whole image. This problem can be limited, but not completely eliminated, by using special anti-reflective coatings on the lens elements. Lens flare proves problematic in lens-based system, as we show later.

Lastly, we have speckle “noise” when the laser is reflected from a rough (even microscopically) surface and interferes with itself, as it will cause the light paths to the sensors to vary in length. Speckle noise results in a granular aspect of the image. While most of the other optical aberrations can be corrected or mitigated, speckle noise is unavoidable with monochromatic light [19,20,21].

The aberrations listed above combine to complicate the analysis of the image and introduce imprecision in the estimation of the true position of the laser in the image. But mitigating these effects requires greater efforts in lens manufacturing, better and more expensive glass recipes [22], apochromatic lens assemblies if one considers using more than one color [23], etc., all of which are inapplicable for inexpensive yet precise range sensors.

If lenses are subject to so many ailments, why not dispense with them? For example, Young [24,25,26], drawing on the works of many other such as Mach [27], shows that if speckle is still present (as it depends mostly on the surface from which the laser is reflected), a pinhole camera eliminates spherical, coma, and other defocusing aberrations, and that it does not introduce field curvature or other distortions in the image. He also shows that pinhole cameras have a theoretically infinite depth of field or, at least in practice, one that is much greater than that of a lens. They can also offer a much wider field of view, in principle up to 180°—it is, however, limited by the thickness of the plate and the diameter of the hole (For plate thickness *t* and hole diameter *d*, the field of view is $2\tan^{-1}\left(d/t\right)$, which is 180° only as t→0 or d→∞ and with an infinite plane for the sensor, which is clearly an infeasible solution. However, pinholes and slits are often beveled on the interior side (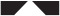) to widen the view angle). A pinhole camera is susceptible to chromatic aberration, but in our case, we can safely ignore this problem, as monochromatic light will be used. The pinhole camera is also susceptible to astigmatism if the pinhole is not perfectly circular (or, if the object is at an angle relative to the optical axis, the aperture will appear as an ellipse), but if mild astigmatism is undesirable, we will show that extreme astigmatism can be favorably exploited.

Indeed, in this paper, we will show that replacing almost all the optical components with a single slit in a laser triangulation range sensor circumvents most optical aberrations, nearly eliminates lens flare, and reduces speckle, thus greatly reducing the need for software correction of aberrations. Furthermore, we will make the case that such a sensor is much simpler and is less expensive to manufacture than conventional lens-based sensors as well as being potentially more accurate.

## 2. Hypotheses

Since a pinhole camera avoids most optical aberrations, offers potentially both a very wide field of view and an infinite depth of field (or, at least, vastly larger than a conventional lens), and reduces lens flare and other internal reflections, we suppose it can be used for a laser triangulation sensor and that we can exploit the diffraction patterns to have better algorithms and better estimation of the laser spot’s position.

**Figure 3:**
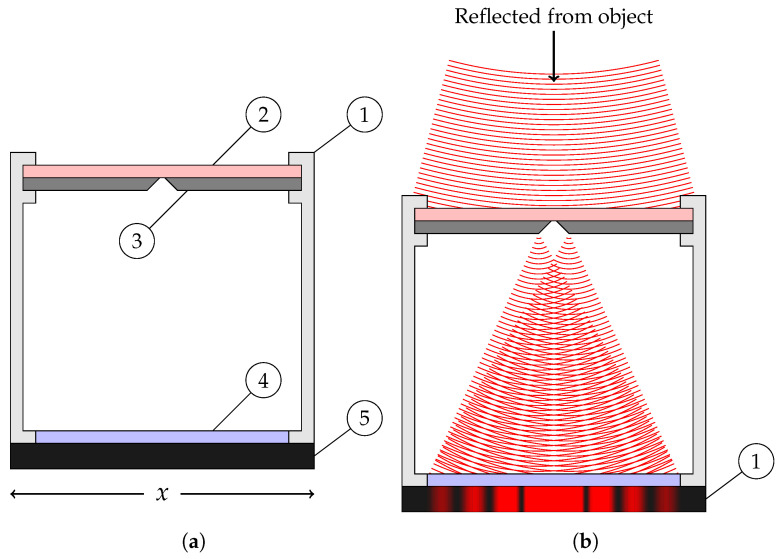
The proposed *camera obscura*. In (**a**), the parts labeled. In (**b**), the diffraction pattern. (**a**) The proposed *camera obscura*. ① Casing. ② Band-pass optical filter. ③ Slit mask. ④ Protective glass. ⑤ Image sensor. Axis indicates the *x* direction relative to the schematic. (**b**) Diffraction pattern created by incident monochromatic light.

The proposed camera configuration is shown in the simplified diagram of Figure 3. The triangulation sensor is composed of only four elements. We find an outer casing, shown as ① in the figure, that holds the band-pass filter ②, the slit mask ③, and the image sensor, shown with its protective window ④ and package ⑤. The actual setup, which will be discussed in the next section, that was built from readily available hardware differs very little in its principles.

**Figure 4:**
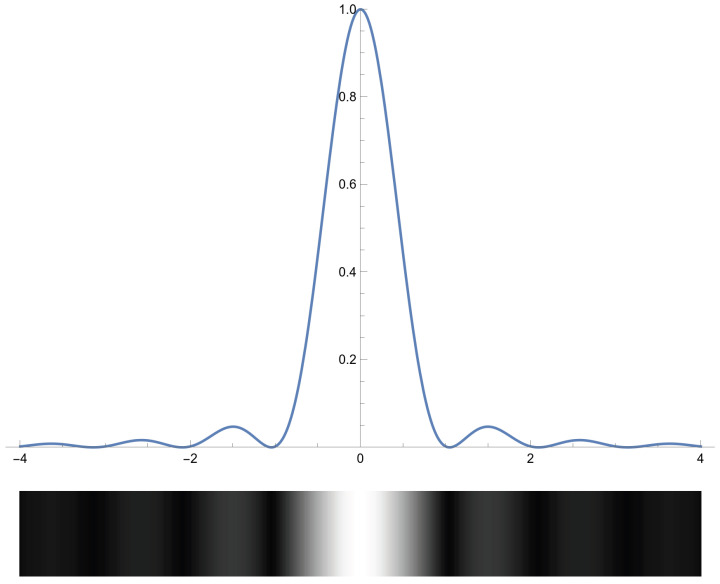
Fraunhofer diffraction (normalized intensity).

The band-pass optical filter blocks all light except for a narrow band of wavelengths corresponding to the laser used. This allows the sensor to operate using essentially monochromatic light. For monochromatic light, the light entering the sensor interferes with itself and creates a specific interference pattern: an Airy diffraction pattern, which is a disk if the aperture is circular [28] or a central bright band with progressively less intense side-bands on each side if the aperture is a slit, as shown in Figure 4 ([29] § 8.5). If the projected laser image takes a pattern of a known or expected form, then we should be able to exploit this knowledge to obtain a better fit on the image and a better estimate of the true center of the spot.

**Figure 5:**
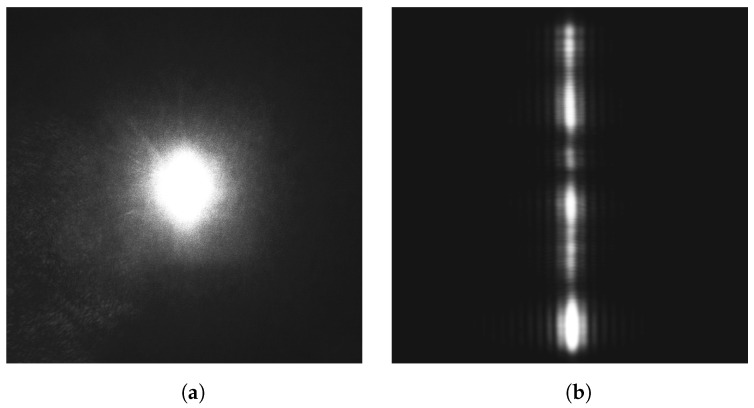
Projection of the laser spot with a lens in (**a**), and a slit in (**b**).

As we mentioned earlier, if pinhole cameras are not subject to as many optical aberrations as lens-based cameras, we still have to worry about speckle noise. Speckle noise originates on the surface of the object from which the laser is reflected. The object surface asperities, whether very rough or minute, will give the reflected light different path lengths to the sensor and therefore different phases, causing it to interfere with itself and give the image a granulated aspect, as shown in Figure 5a.

If the general shape of the projected image is known, we can use a better algorithm than a simple centroid to find its center. The models considered are the simplified Gaussian function (not to be confused with a Gaussian distribution) and the Fraunhofer diffraction formula: an approximation of the Airy diffraction pattern. Once a good estimate of the center of the projected image is obtained, we can translate from the pixel space to the actual distance. One possible way to do so is to use a geometric model that takes into account the various parameters of the sensor and translate the projection center *x* to the distance *d*, just as shown in Figure 1. Another way would be to place the sensor on a high-precision linear displacement table and to acquire a number of laser point reflections from a target at known positions. These points are to be stored in a look-up table in the sensor, and a new position *x* in the pixel space can be searched for in the table and the distance *d* interpolated from neighboring known values in the table. This second method seems suboptimal, but it may be preferable as it takes into account any deviation from the ideal device due to manufacturing.

To reduce the effect of speckle, we use a vertical slit (relative to the horizontal axis of the image sensor) rather than a circular aperture. This can be seen as a special case of astigmatism, where in one direction (along the slit), the focal length is very large, and along the other (across the slit), the focal length is short. This causes a stretch in the reflected laser image, as shown in Figure 5b. In this way, speckle noise is spread mostly vertically, allowing a better horizontal, line-by-line, analysis of the image. We also suppose that the projection angle (α in Figure 1) is moderate so that the projected image, including the interference pattern, remains approximately symmetrical [30].

Therefore, our main hypothesis is that replacing a lens with a slit improves the accuracy of a laser triangulation sensor. The use of a slit circumvents most of the optical aberrations found in lenses, reduces the influence of speckle noise, and aids image analysis by creating (mostly) symmetrical diffraction patterns. Symmetrical diffraction patterns could be amenable to simpler or less computationally expensive image analysis algorithms. As a side effect, we surmise that using a slit will significantly reduce the mechanical complexity and cost of manufacturing laser triangulation sensors as well as potentially making them more robust and easier to adjust and calibrate.

## 3. Methods

To create a useful data set, we built a precision position table to move with accuracy a target on which to reflect the laser and to support instruments such as the table’s controller, the laser source, and the camera. The table is fully automated under the control of a standard PC and over a serial/USB cable.

**Figure 6:**
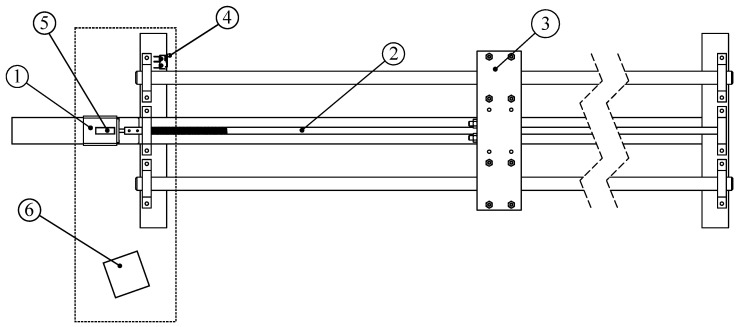
A simple high-precision position table. Useful range: 150 cm. ① Step motor, ② worm gear, ③ target carriage, and ④ limit switch. On a metal plate (dashed): ⑤ laser, ⑥ camera with lens or slit.

The precision positioning table is shown in Figure 6. It is controlled by a WantMotor 32BYGHm809 (Jiangsu Wantai Motor Co., Ltd., Changzhou, China) precision step motor capable of 6400 steps by revolution (shown as ① in the figure), a worm gear ②, and a carriage ③ on which we can place different materials. The useful range of the table is 150 cm, starting at the limit switch, shown as ④. Since during acquisition the carriage was only moved in one direction (away from the camera), there was no kickback, as the worm gear only pushed against the carriage. The displacement error after 60,000 steps was well under 1 mm, which gives us displacements of 25 μm ± 0.017 μm per acquisition.

The laser is a Class IIIa 650 5 mW 5 V TTL red laser diode, and it is placed over the step motor and in the same orientation as the worm gear; the laser is in the figure.

The camera used, shown as ⑥ in the figure, is a C-Mount Sentech STC-MBS231U3V USB3 camera. The camera has a resolution of 1920 × 1200 pixels over a 7.04 mm × 11.3 mm CMOS full-shutter image sensor. The camera uses a Sony IMX249 monochrome image sensor, which produces a gray-tone image and therefore avoids any artifacts that would result from a Bayer color filter sensor. The maximum frame rate for the camera at full resolution is 41.6 fps, but the camera was used in snapshot rather than in movie mode. The camera was placed 30 cm away from the laser and angled so that its optical axis crossed the worm gear.

For the images themselves, we produced for each material two sets of images: one using a lens and another using a slit. For image acquisition with a lens, a Computar 50 mm fixed focal length, *f*/1.8, C-mount lens was used, with the iris set at f/12 to have image illumination comparable to that of the slit. For the images acquired with a slit, we used a 25.4 mm-diameter slit disk compatible with the C mount 50 mm tube and with a 200 μm × 3 mm slit-centered slit.

The materials used for the tests were chosen to be representative of textures likely encountered in primary- or secondary-sector processes or manufacturing. The materials chosen were brushed metal; unevenly rusted metal; light-colored, planed but unvarnished wood; standard white printer paper; black and reflective PVC (black electrical tape); and microfiber fabric.

The acquisition strategy was straightforward. For each configuration, we started the carriage at the beginning of the useful range of the sensor: that is, when the laser spot happened to be fully captured. This corresponded to 60 cm away from the carriage when it was pushed against the limit switch (④ in Figure 6). The slit field of view is naturally wider than that of the lens, but both begin at 60 cm from the limit switch. We then proceeded to acquire five independent 1920 × 600 images (with the region of interest centered vertically in the 1920 × 1200 complete image); then we moved the carriage forward—to the right relative to Figure 6—by 25 μm. The process was repeated until it reached the end of the sensor’s range, (where the spot stops being visible), 102 cm away from the limit switch, which amounts to 16,800 × 5 captures. This process was done for each pair of material and lens or slit combination.

In addition to lens, slit, and other aberrations, the image sensor itself is noisy. For a CMOS image sensor, there are two type of noise. The first one is thermal noise that makes each pixel value change randomly. It is usually supposed to be small and independently and identically normally distributed. The second type of noise is the black threshold, where pixels report values much higher than zero despite not being exposed. We consider this black threshold as a shared systematic bias: while individual pixels might be “hotter” than others, we suppose they all have the same bias.

This bias should therefore be removed before proceeding to further image analysis to find the center of the reflected laser spot. The black threshold, *t*, can be estimated once at calibration, or it can be estimated for each image. To estimate the black threshold for an image, we average all pixels of the image except for the region containing the reflected laser spot. The reflected laser spot position is first estimated coarsely using the centroid, or the center of mass of the image. That is, for a n×m pixel image, the centroid is given by

(2) $$c=\frac{\sum_{i=1}^{n}\sum_{j=1}^{m}y_{i,j}\left(i,j\right)}{\sum_{i=1}^{n}\sum_{j=1}^{m}y_{i,j}},$$

where $y_{i,j}$ is the intensity of the pixel at coordinates (i,j) and is understood to be a two-dimensional vector. The centroid is then used as the center of a 320×600 pixel exclusion region for the lens and a 128×600 pixel exclusion region for the slit.

Once *t* is estimated, we correct the pixel values to compensate for the black threshold without renormalization: that is,

$$y_{i,j}^{\prime }=\max\left(0,y_{i,j}-t\right)\;.$$

We are now ready to find the center of the reflected laser spot. We have two distinct cases to consider: one where the spot is projected by a lens and is approximately circular, as shown in Figure 5a, and another where the spot is projected through the slit, as shown in Figure 5b.

Ross describes some typical ways to find the center of the reflected laser spot projected by a lens [31]. We chose a simplified Gaussian function:

(3) $$A_{max}\;e^{-{∥r∥}^{2}},$$

where $A_{max}$ is the maximum amplitude at the center, and *r* is a linear transformation applied to the coordinates of the image plane:

(4) $$r={\left[\begin{array}{cc} \sigma_{xx} & \sigma_{xy}\\ \sigma_{yx} & \sigma_{yy} \end{array}\right]}^{-1}\left[\begin{array}{c} x-\mu_{x}\\ y-\mu_{y} \end{array}\right].$$

The amplitude $A_{max}$; the covariances $\sigma_{xx}$, $\sigma_{xy}$, $\sigma_{yx}$, $\sigma_{yy}$; and the means $\mu_{x}$ and $\mu_{y}$ are estimated using least mean squares regression. The values of $\mu_{x}$ and $\mu_{y}$ give us the center of the reflected laser spot.

**Figure 7:**
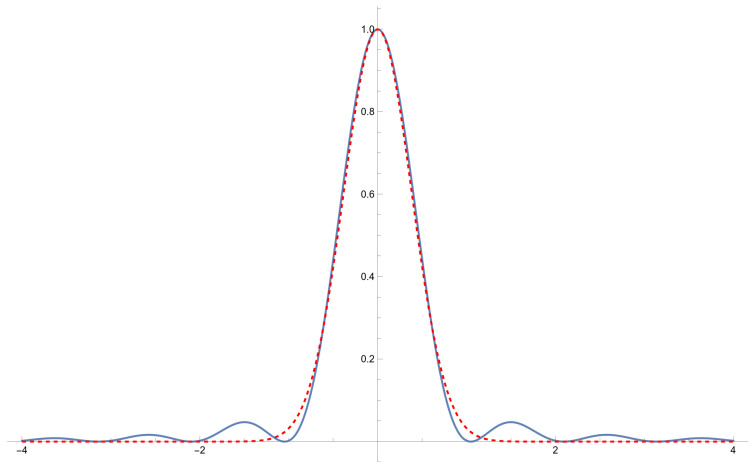
Fraunhofer diffraction (blue) approximated as a Gaussian (dashed red).

For the images captured using the slit, we proceed via a line-by-line analysis by fitting a one-dimensional Gaussian function:

(5) $$A_{max}\;e^{-{\left(s(x-x_{0})\right)}^{2}}.$$

where $x_{0}$ is the center, *s* contains the wavenumber and other scalings, and $A_{max}$ is the maximum amplitude. This approximation is quite reasonable, as shown in Figure 7. The side lobes in the Fraunhofer diffraction formula, Equation (6), vanish rapidly, and the Gaussian function, Equation (3) (or Equation (5)), very closely matches the central peak.

We see from Figure 7 that a simplified Gaussian is a good approximation of the actual Fraunhofer diffraction formula given by

(6) $$F\left(x\right)=A_{max}{\mathrm{sinc}}^{2}\left(s\left(x-x_{0}\right)\right).$$

The Fraunhofer formula, however, should be much closer to the actual projection being observed and, once fitted, is potentially a much better estimate of the actual center of the reflected laser spot, but using the simplified Gaussian of Equation (5) is less computationally intensive for the sensor’s onboard processor.

The final slit center is estimated as the average of all line-by-line centers, which are either estimated by the simplified Gaussian or by the Fraunhofer diffraction formula. These averages yield good estimates of the horizontal position of the reflected laser spot. Indeed, for the images captured using the slit, the projections only move horizontally as the laser and the optical axis are in a same plane parallel to the *x*-direction of the image sensor.

Once *x* is estimated by either the centroid, the simplified 2D Gaussian for images captured with a lens, or by the line-by-line average using simplified Gaussian or Fraunhofer’s formula for images captured with the slit, we use Equation (1) to estimate the distance to the sensor. However, Equation (1) contains a number of parameters that must be found. For example, to use Equation (1) using a slit and Fraunhofer’s formula, we use all the images from a data set captured using a slit and estimate all the *x*s using Fraunhofer’s formula; we then use these *x*s and their corresponding known positions to fit the parameters of Equation (1) using a modified least mean squares approach [32]. The errors reported in Section 4 are the differences between the positions predicted from Equation (1) and the known positions.

## 4. Results

In this section, we will present the results from the experimental setup described in the previous section. We will show that the positions estimated by the fit of the simplified Gaussian function on the reflected laser spot projected by a lens are not as precise as the positions estimated by both the fit of the simplified Gaussian function and by the fit of the Fraunhofer diffraction formula over the reflected laser spot captured using a slit.

First, let us discuss the translation from an estimation of the center *x* to the distance *d*, as given by Equation (1). If $d_{0}$, *u*, *b*, and *f* are known with high certainty, the translation is done quite easily. If they are not known, or are not has been retained. known with great precision, we can use the data and fit Equation (1) on the data to find the parameters ${\hat{d}}_{0}$, $\hat{u}$, $\hat{b}$, and $\hat{f}$ that best fit the observations. This allows us to assess the quality of the estimates on the positions of the reflected laser spot, whether through a lens or a slit.

**Figure 8:**
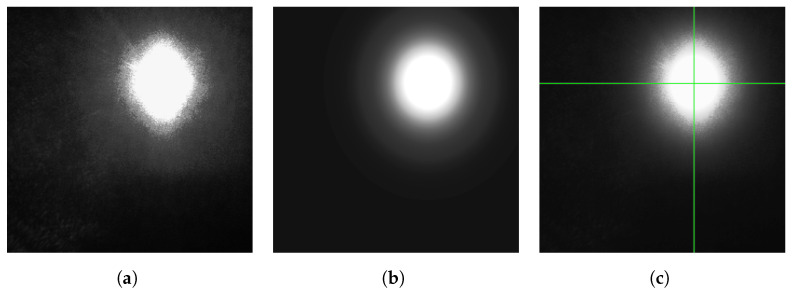
Analysis of a laser spot through a lens. (**a**) Image cropped around detected reflected laser spot. (**b**) Reflected laser spot modeled as a Gaussian. (**c**) Reflected laser spot with center superimposed.

Let us now present the results obtained from a sensor using a lens. First, we examine its behavior and properties. In Figure 8a, we observe the cropped reflected laser spot through a lens. We see that it is diffuse, despite being in focus, and that it exhibit conspicuous, but as remarked earlier, unavoidable, speckle noise. We also see that the spot is not perfectly circular but is elongated. This is the effect of the laser beam not being perfectly circular when interacting with the target material: in this particular example, white printer paper. In Figure 8b, we see the best-fit Gaussian obtained from the raw data. From these fit parameters, we obtain the estimation of the center of the spot, shown in Figure 8c as a green cross.

**Figure 9:**
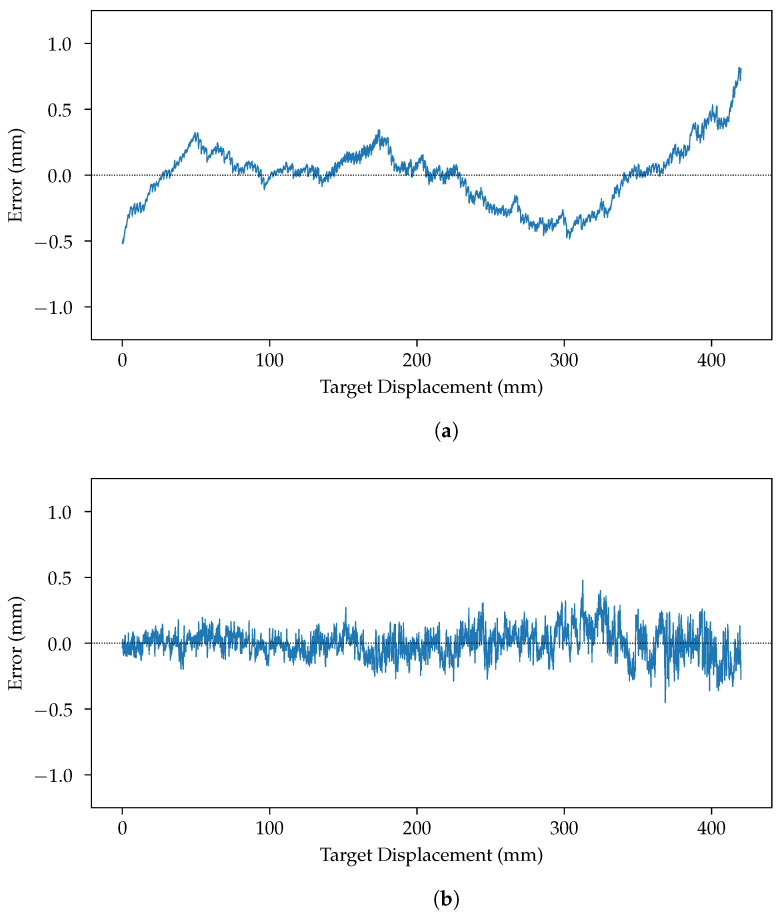
Error in center estimations from both methods. The material measured in this figure is the brushed metal. (**a**) Error in center estimation from using a lens. (**b**) Error in center estimation from using a slit.

In Figure 9a, we see the errors in mm between the estimated positions using a lens and the true position. The position of the reflected laser spot is estimated for each measurement. It is then translated into the actual distance using Equation (1), with its parameters estimated on the whole data set. We see that the predicted distances are precise but not very accurate. At both the far left and far right, the readings are thrown off by optical aberrations. The variation near the center is caused by lens flares, which are more visible as the laser spot approaches the center of the image.

**Figure 10:**
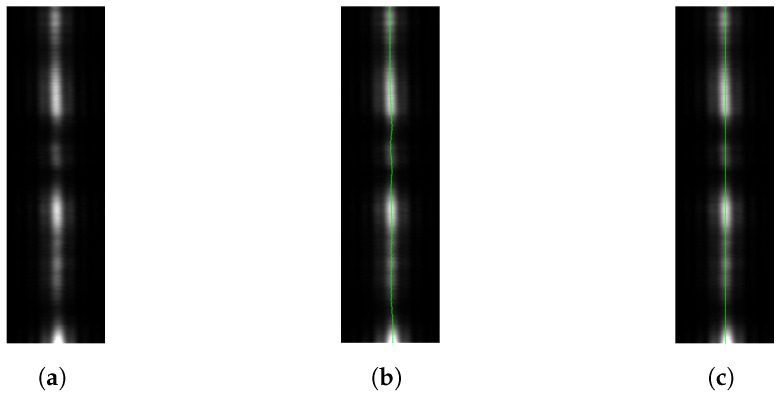
Reflected laser spot through a slit. (**a**) Image cropped around detected reflected laser spot. (**b**) Reflected laser spot with centers, line-by-line. (**c**) Reflected laser spot with center superimposed.

**Figure 11:**
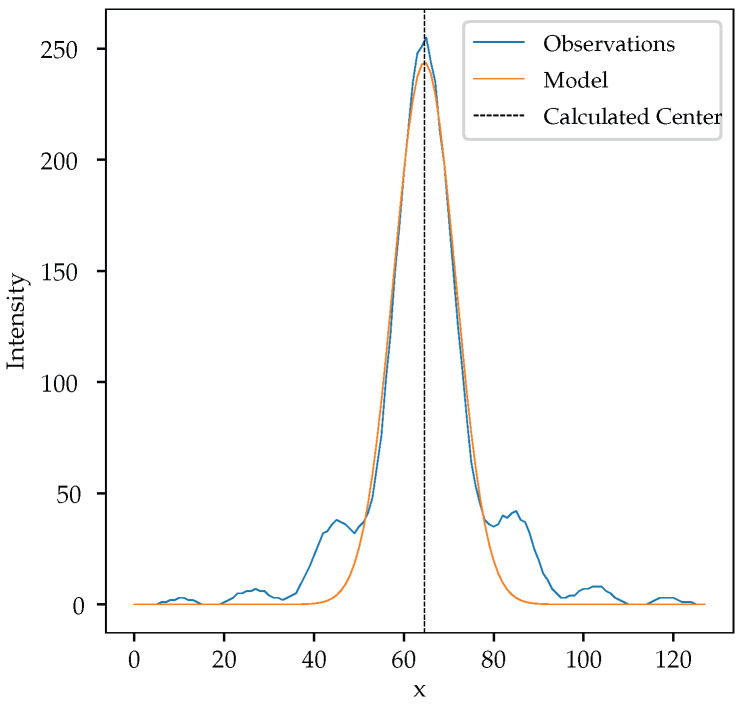
Gaussian fitted to a single-line observation.

The process is quite similar when using a slit. In Figure 10a, we see the cropped image of the isolated reflected laser spot. For each line, a Gaussian is fitted, as is shown in Figure 11, and the center is estimated for that line. We see all the line-by-line centers found in Figure 10b. These centers are averaged to obtain the final estimate of *x*, which is the horizontal position of the spot as projected through the slit. The process is similar (but not shown in the figures) for the fit using Fraunhofer’s diffraction formula.

**Figure 12:**
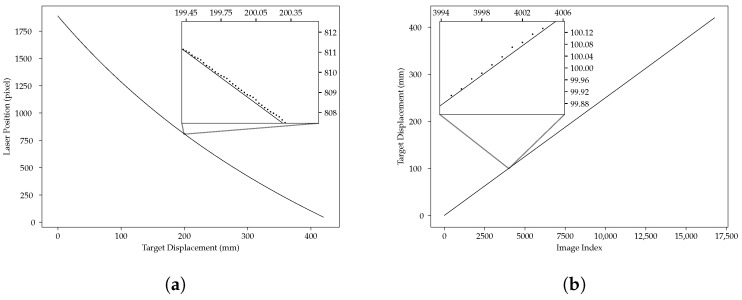
Geometric model, fit, and positions. (**a**) Geometric model fitted on slit data. Dots are estimated centers from image analysis, and the straight line is Equation (1) fitted to the data. (**b**) Known positions vs. fitted geometric model. The dots are known (discrete) positions, and the line is the prediction from the geometric model.

Figure 12a shows the result of the estimation of the different parameters of Equation (1) fitted on all the centers found using all the centers estimated using the slit for the white printer paper data set. Figure 12a shows that the curve does not go through every point—as it could not, since Equation (1) is rather constrained—but that, on average, the errors are very small. Figure 12b shows the discrepancy between known positions (obtained during the target displacement by the precision position table) and the prediction from Equation (1) with its parameters estimated from the computed centers. Lastly, Figure 9b shows that the slit is more accurate and more precise than the lens. It shows that the sensor using a slit is not affected by flares and so, on average, is closer to the real value than the sensor using the lens.

**Figure 13:**
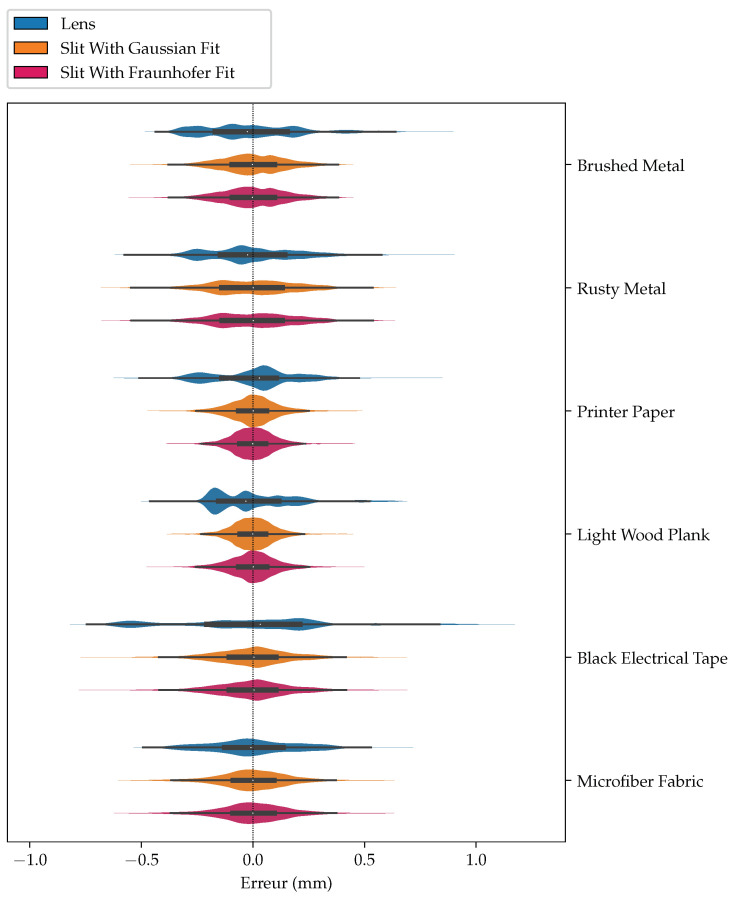
Violin plots of errors for the lens compared to the slit. The *x*-axis shows the magnitude of errors (dashed line is zero), and the *y*-axis is the normalized probability.

Table 1 compares the maximum error (MAX) and the mean average error (MAE) for the lens, the slit with a Gaussian fit, and the slit using Fraunhofer’s diffraction formula for the chosen materials. In all cases, the slit methods are better than the lens method, and sometimes significantly so. This also corroborates our interpretation that using a slit leads to results that are both more precise and more accurate than when using a lens. This interpretation of the results is also confirmed by the violin plots of Figure 13. Violin plots are an extension of plots wherein probability densities are superposed to quartiles. From Figure 13, it is apparent that the distributions of errors from the slit (in tan ■ for the Gaussian function and in rose ■ the Fraunhofer diffraction formula) are all more compact than the distributions of errors from the lens (in slate ■). We also remark that the error distributions from the lens are multi-modal, while this effect is much less present with the slit methods, as the optical aberrations, being almost eliminated, do not perturb the estimation as much.

## 5. Discussion

The results seem to indicate that using a slit in a laser triangulation sensor is a promising avenue. Indeed, Figure 9 shows that a laser triangulation sensor using a slit is less subject to various optical aberrations: in particular, lens flare. The results also show that, on average, the sensor using a slit is both more precise and more accurate than the sensor using a lens.

However, the proposed method is not without flaws. First, it depends on the wavelength of the laser. In our experiments, we chose a Class IIIa 650 nm red laser both out of convenience (as it was readily available) and safety (as it is mostly harmless), but a shorter-wavelength laser could be used. The spread of the diffraction pattern is strongly linked to the incident wavelength; a shorter wavelength would yield both a more compact diffraction pattern and finer-grained speckle, which, if fine enough, could be averaged within pixels by the CMOS sensor itself. Second, we could use a more powerful laser, which would counteract the relative “darkness” of a camera using a slit aperture. For the tests, we had the luxury of higher exposure times to gather enough light to form images clear enough for analysis, but some applications might necessitate a great number of readings per second and, therefore, very short exposure times. Additionally, if greater distances are considered, the wavelength and power can be chosen to accommodate the specific application considered [33]. Thirdly, using simplified functions (such as the simplified Gaussian or the Fraunhofer diffraction formula) certainly deprives us of the better fit we might obtain from a formula such as the Fresnel diffraction formula. Indeed, better exploiting information in the diffraction pattern could not only give us a better estimation of the position of the maximum but also give us information about the angle of incidence, as the diffraction pattern is approximately symmetric only for small angles. Better estimation algorithms will be the subject of future work. Lastly, precision is affected by the materials measured. Rough, porous, or irregular specular reflection materials prove difficult to measure accurately—regardless of the laser spot acquisition method.

The simplified hardware required is also quite interesting. In both systems—lens and slit—we find a band-pass optical filter that conveniently filters out undesired light sources and an image sensor—typically CMOS. But a slit, even precision-manufactured, is much less expensive, and resource-consuming than a lens, especially since “a lens” is rarely just one piece of glass: it is more often a rather complicated assembly comprising many lenses that are designed to compensate for all kinds of optical aberrations [34,35]. Furthermore, as we showed that the reflected laser spots only travels horizontally in the field of view, we might not need a full sensor, and a line-scan sensor (which, despite the name, typically includes more than one row of pixels) could suffice. Such an image sensor would also reduce the cost and size of the laser triangulation sensor.

The image analysis algorithms are also likely to be simpler when we explicitly exploit the shape of the diffraction pattern. In the case of a full two-dimensional Gaussian, we either must explicitly and directly estimate the covariance matrix Σ and compute its inverse (as in Equation (4)) using the usual estimation method, $\Sigma =\frac{1}{n}\left(XWX^{T}-n\vec{\mu }{\vec{\mu }}^{T}\right)$, with *W* being the weights (pixel intensities), *X* the column–vector matrix of pixel coordinates, and $\vec{\mu }$ the average coordinate—the centroid—or we use some other regression framework: for example, L-BFGS-B, which was used for our experiments in [36,37,38,39]. For a one-dimensional fit, the estimation of the center and spread is much simpler, and each line could, at least in principle, be processed in parallel.

We are now confident that the method can be exploited for laser triangulation sensors.

## 6. Conclusions

The starting hypothesis was that using a slit instead of traditional lenses in a laser triangulation sensor could improve the accuracy of the sensors by removing the optical aberrations inherent to lenses, and that a slit could be amenable to simpler analysis algorithms—or at least algorithms that are less computationally expensive. We have shown that line-by-line analysis of the diffraction pattern of a reflected laser spot through a slit is not only simpler but gives more accurate results than the image analysis of a reflected laser spot through a lens, and that this was true for both fit methods (simplified Gaussian and Fraunhofer’s diffraction formula) and for all considered test materials.

Further work is of course considered. Preliminary testing (not shown in this work) seems to indicate that the gain from using the Fraunhofer diffraction formula to find the center is negligible compared to the simple Gaussian approximation, but we intend to explore more complex models of the diffraction pattern, especially to take the asymmetry arising from larger incident angles into account.

## Figures and Tables

**Table 1:** Compared measurement errors for different materials.

	Lens		Slit with Gaussian Fit		Slit with Fraunhofer Fit	
Material	MAX μm	MAE μm	MAX μm	MAE μm	MAX μm	MAE μm
Brushed Metal	851	182	520	115	527	155
Rusty Metal	860	164	640	158	638	158
Light Wood Plank	651	151	429	72	476	81
Printer Paper	812	144	468	81	437	72
Black Electric Tape	1098	280	735	134	743	134
Microfiber Fabric	677	154	602	118	600	119

## Data Availability

Captured image data are not hosted publicly but can be requested by contacting the corresponding author. The requester should provide about 1 TB of storage in order to receive the complete data set.
